# Modifications to the delivery of NHS face-to-face general practice consultations during the COVID-19 pandemic in England

**DOI:** 10.12688/f1000research.52161.1

**Published:** 2021-03-31

**Authors:** Lorna J. Duncan, Kelly F.D. Cheng

**Affiliations:** 1 Centre for Academic Primary Care, Population Health Sciences, Bristol Medical School, University of Bristol, Bristol, UK; 2 Bristol Medical School, University of Bristol, Bristol, UK

**Keywords:** COVID-19, SARS-CoV-2, coronavirus, general practice, primary care, face-to-face consultation, delivery model, transmission

## Abstract

**Background: **To minimise transmission of SARS-CoV-2, the virus causing COVID-19, delivery of general practice consultations has been modified to enable the separation of diagnosed or suspected COVID-19 patients from others. Remote triage and consultations are currently the default model, with adapted face-to-face contact used when clinically necessary. This study aimed to identify the modified face-to-face delivery models used across England, and evidence for their effectiveness.

**Methods: **In June 2020, a national survey was sent by email to the 135 Clinical Commissioning Groups (CCGs) in England to identify local organisation of face-to-face general practice consultations since March 2020. An email was sent to Public Health England (PHE) requesting information about COVID-19 outbreaks or clusters linked to general practice.

**Results: **All CCGs responded. Separation of COVID-19 patients from others was achieved using combinations of the following models:

1. zoned surgeries (used in 47% of CCGs), where COVID-19 and other patients are separated within their own practice;
2. ‘hot’ or ‘cold’ hubs (used in 90% of CCGs), separate sites where COVID-19 or other patients registered at one of several collaborating practices are seen;
3. ‘hot’ and ‘cold’ home visits (used in 70% of CCGs).

One of seven model combinations was used across each CCG, with some flexibility according to changing need shown through hub availability. Concomitant PHE data showed less than 2% of COVID-19 outbreaks or clusters in England were linked to general practice.

**Conclusions: **Varied, flexible ways of delivering face-to-face general practice consultations were identified. While COVID-19 outbreaks or clusters linked to general practice constituted a small proportion of totals, their investigation, together with evaluations of the modified delivery models in terms of management of COVID-19 and other conditions and impacts on staff and patients, may aid future management of the pandemic and identify aspects of adapted practice of benefit beyond this.

## Introduction

While more than 80% of patients with COVID-19 may not require hospitalisation, ^1^ many will seek treatment in general practice. In order to minimise transmission of the causative severe acute respiratory syndrome coronavirus 2 (SARS-CoV-2) during general practice (GP) consultations, NHS England’s Standard Operating Procedure was revised in March 2020 to a remote triage and consultation default, with adapted models for face-to-face contact when clinically necessary. ^2^ The use of telephone, video and online consultations in English general practice has been studied elsewhere. ^3^ In this paper we report on the delivery of face-to-face general practice consultations during the pandemic, largely focussed on the spring and summer of 2020.

When, after remote triage, a face-to-face general practice consultation is considered necessary, the requirement to separate those patients with suspected or diagnosed COVID-19 [‘COVID-19’ patients] from others is clear. Protection must be afforded to all on-site and NHS guidance suggests two possible ways to manage patients, premises and workforce for optimal infection prevention and control (IPC), alongside dedicated home visiting services for those shielding: ^2^

(i) Zoned practices: In this model, patient cohorts are separated within their own practices. Designated areas e.g., ‘red’ and ‘green’ zones, may be used to manage COVID-19 and other patients, respectively. Careful management is needed to minimise cross-contamination between groups, including separate walkways and consultation rooms, and staff allocated to one zone only. Zoning may therefore be impractical in some surgeries.
(ii) Hot and cold hubs: A primary care hub may be designated as either ‘hot’ or ‘cold’, to treat COVID-19 or other patients respectively. It is available to patients registered at one of several collaborating practices. Where hot hubs are sited separately to non-COVID services, IPC procedures may be more straightforward than in zoned practices. ^2, 4^
(iii) Dedicated home visiting: Home visiting services, modified to minimise cross-contamination, are necessary for those patients unable to access other face-to-face services, or where such provision is otherwise considered appropriate during the pandemic. Staff work exclusively with COVID-19 or other patients, and the number of activities undertaken during visits should be maximised to limit the requirement for additional face-to-face consultations. This service may be organised collaboratively, such as across Primary Care Networks, or by individual practices.

NHS guidance indicates that decisions regarding model use should be determined locally, in agreement with the relevant Clinical Commissioning Group (CCG) responsible for planning and commissioning NHS health care services in the area. It also recognises that flexible models may be required as patient demand and workforce capacity fluctuates throughout the pandemic. ^4^

This study aimed to identify the ways in which delivery of NHS face-to-face general practice has been re-organised across England to meet IPC standards during the pandemic, together with any evidence regarding the effectiveness of these measures.

## Methods

### 1. CCG survey

A cross-sectional survey of the 135 CCGs in England was conducted to identify how face-to-face general practice consultations were being delivered across the country.

Survey design

Survey questions were devised by the study team. They concerned models of face-to-face consultations in use and the patient populations each were available to; prior use of the hub model; and planned evaluations. Questions were pre-tested with a researcher experienced in survey design, two CCGs and one provider of primary healthcare. Minor changes to wording were made for clarity. The final questionnaire is available as *Extended data*. ^13^

Data collection

Questions were sent by email to all CCGs in June 2020 under the Freedom of Information (FOI) Act 2000. This legislation enables public access to recorded information held by public authorities in England. Responsibility for cleansing data lies with the authorities responding to FOI requests ^5^ and research ethics approval was not required.

Individual CCGs were identified on the NHS England website and their specific FOI procedures were followed. FOI regulations mandate a response timeframe of 20 working days. Where, rarely, replies were not received within this time, follow-up emails were sent, and telephone calls made if necessary. All responses were collated in an Excel spreadsheet for analysis.

Data analysis

Data was extracted for each survey question, with any queries regarding response interpretation discussed between the authors. Email and telephone communication with CCGs was also used occasionally for clarification or updates. Numeric and narrative analyses were undertaken for responses to each question. Different face-to-face delivery models were identified, and each CCG was assigned to a model combination of best fit for nationwide comparison.

### 2. Public Health England (PHE) query

Specific data on clusters or outbreaks of acute respiratory infections with at least one linked case testing positive for SARS-CoV-2 are recorded for hospitals and care homes in the weekly COVID-19 surveillance reports *,* published by Public Health England (PHE). ^6, 7^ [‘Clusters’ are cases linked to a setting through surveillance testing; ‘outbreaks’ are reported directly from settings]. To obtain similar information regarding general practice, an email was sent to PHE in December 2020, under the FOI Act 2000. The questions asked are available as *Extended data*. ^14^

## Results

### 1. CCG survey

Responses

Replies were received from all CCGs, 99% in June and July 2020, with the final response received on 2 ^nd^ October.

Response interpretation

Terminology used in responses varied – ‘hot sites’, ‘hot clinics’ and ‘resilience hubs’ could be used to refer to the same service for example. Colour-coding, e.g., ‘green’ and ‘amber’ sites, could also be used differently, and service provision for non-COVID patients was not always clear. As a consequence the distinction between hot hubs, cold hubs and zoned practices was not always immediately evident. Complete response sets, including any additional documentation provided on model pathways and usage data, were therefore used to interpret and categorise all face-to-face consultation types according to the models in this paper.

The following interpretation of the data is the authors’ own and has not been approved or otherwise by the CCGs.

Adapted delivery models

**Figure 1.**
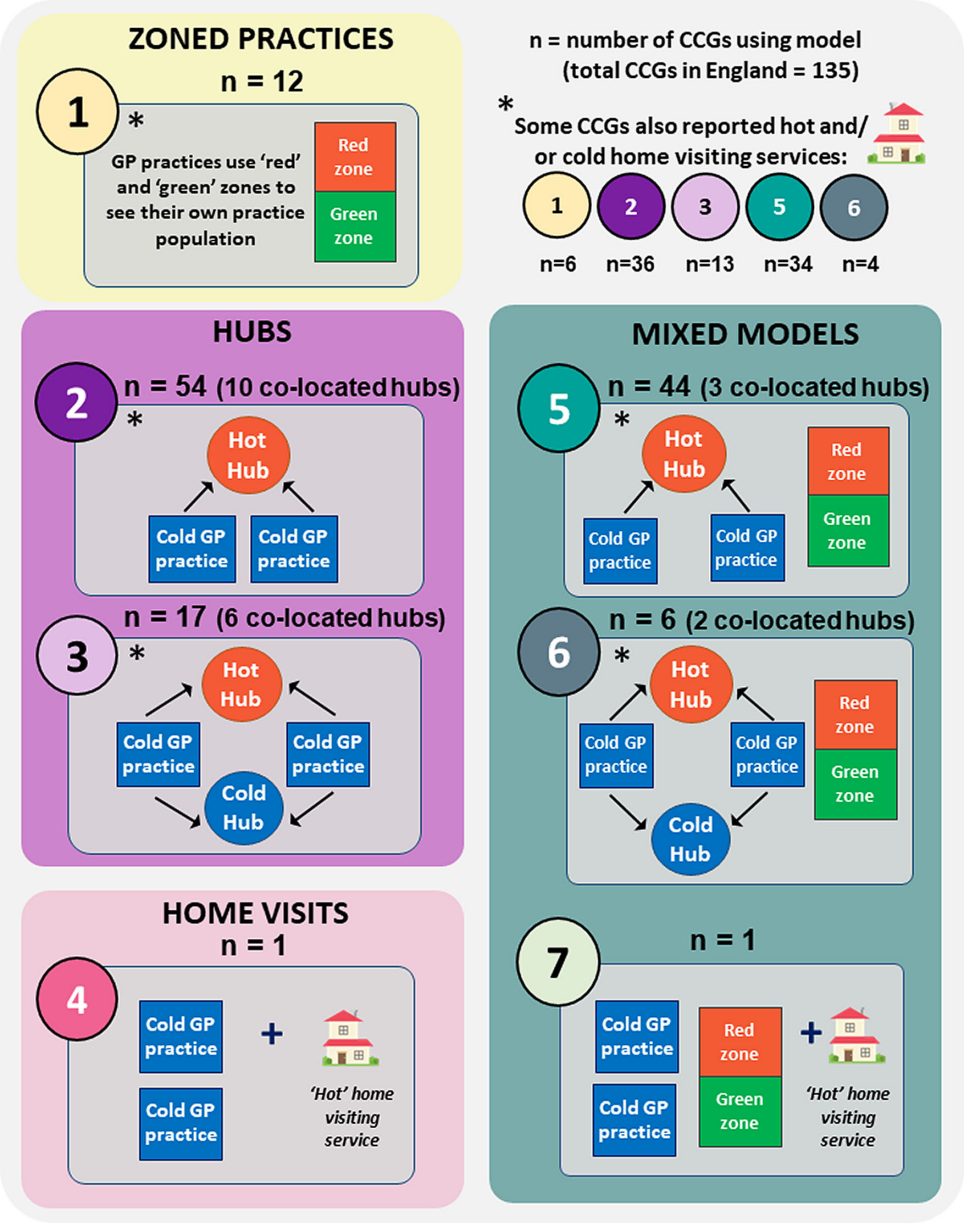
Models used to separate COVID-19 and other patients during face-to-face NHS GP consultations in England. Authors’ interpretations of CCG responses according to the following definitions:
•Zoned practice: co-location of hot and cold services on a single site, serving own practice list•Hot or cold hub: site of multi-practice working for COVID-19 or other patients respectively

General practice face-to-face delivery has been modified in each CCG using combinations of the three models previously described:

(i) Zoned practices (model 1 in Figure 1), available to the entire patient populations served, were reported by 63 (47%) of CCGs. All but 12 of these also used hubs at the time of reporting. Most commonly, two closed ‘red’ and ‘green’ areas with different entrances and exits were used. Rarely, cohorts could also be separated temporally, with COVID-19 patients alone seen at certain times. The latter model was described in updated NHS England guidance (version 2, dated 5 ^th^ April 2020) for surgeries where provision of separate spaces for different cohorts was not possible. ^2^
(ii) ‘Hot’ or ‘cold’ hubs (models 2 and 3 in Figure 1), were reported by 90% of CCGs. All of these had hot hubs, with 17% also using cold hubs. Hubs were generally available to all of the combined patient populations served. Occasionally however, cold hubs had more specific uses - a ‘super-green’ hub for patients requiring additional shielding for example and a ‘purple’ hub for routine treatments including vaccinations and maternity checks. Hub reach extended from several practices to entire CCGs. They were sited in re-purposed buildings (surgeries for example, or hubs that usually offered extended GP access), a racecourse, temporary structures (e.g., portacabins and marquees) or provided as drive-through facilities. Most CCGs indicated they had used hubs prior to the pandemic, mainly for the provision of extended hours GP access for patients.
  The use of ‘co-located’ hubs was indicated in 21 CCGs, whereby hot hubs were sited adjacent to cold hubs (n=4) or cold practices (n=17).
(iii) ‘Hot’ and/or ‘cold’ home visiting services, were specified by 70% of CCGs - 15% for hot appointments only, 10% for cold and 45% for both cohorts. These usually served patients unable to access other face-to-face services but were occasionally the main form of COVID-19 service provision. Delivery could be provided by practices, collaborative networks or CCG acute visiting services, and in some cases operated out of hubs. A small number of CCGs offered home monitoring of COVID-19 patients, via pulse oximetry for example, as an additional service.

The different means of delivering face-to-face services were compared for each CCG. Overall approaches taken were found to fit one of seven model combinations, albeit with some distinctions, notably the different usage of home visits and co-located hubs.

**Figure 2.**
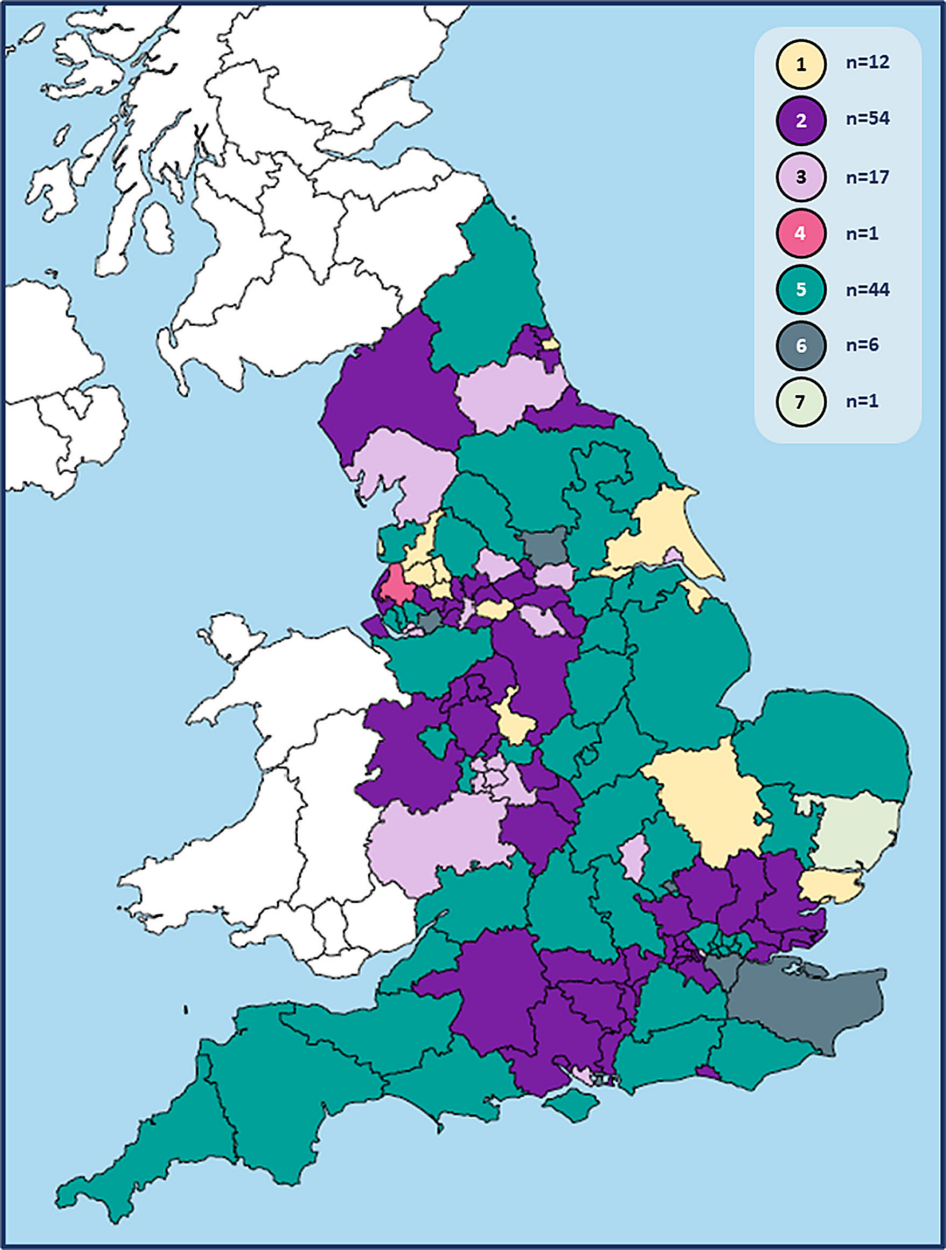
Local models used to separate COVID-19 and other patients during face-to-face GP consultations across England. Model combinations:* 1: zoned practices (+/- home visits) 2: hot hubs + cold practices (+/- home visits) 3: hot hubs, cold hubs + cold practices (+/- home visits) 4: hot home visits + cold practices 5: hot hubs, cold hubs + zoned practices (+/- home visits) 6: hot hubs, cold hubs, zoned practices + cold practices (+/- home visits) 7: zoned practices, cold practices + hot home visits. *Authors’ interpretations of CCG responses according to the following definitions:
•Zoned practice: co-location of hot and cold services on a single site, serving own practice list•Hot or cold hub: site of multi-practice working for COVID-19 or other patients respectively N.B. 15 CCGs did not describe face-to-face consultations for ‘cold’ patients. 14 of these were assigned to model combination 2 as hot hubs were described which were not co-located with cold services; and 1 was assigned to model combination 5. The face-to-face delivery data we have presented was correct between March 2020 and the date of response [June/July (n=134) and October (n=1) 2020].

The seven model combinations are illustrated in Figure 1, and their distribution among CCGs shown in Figure 2. The diverse spread of models and size of CCGs seen in Figure 2 reflect the varied geography and population across England. A degree of cross-CCG working was evident in replies, and a small number of CCGs had access to hubs across neighbouring boundaries.

Evaluations and flexible models

87% of CCGs reported on-going, complete or intended reviews, generally of hub and/or telephone triage use, although one CCG was considering the potential of its drive-through model for influenza vaccinations, and others were intending to consider staff or patient perspectives. In total 23 CCGs reported using reviews to facilitate dynamic models, with hubs either available but as yet unused (n=3) or having been used initially and then stood down (n=20). [Assignment to model combinations 1-7 was based on provision at the time of CCG reporting.] More recent communication (Oct 12th-16th 2020) with three such CCGs using the zoned practice model (#1) confirmed that, despite COVID-19 incidence requiring local lockdowns at that time, ^6, 7^ GP escalation plans had not yet been necessary although hub sites remained available. Some other CCGs also indicated lower use of COVID-19 face-to-face consultations than anticipated.

17 CCGs provided data on face-to-face contact across 21 hot hubs. While representing only a small proportion of total hubs, wide variations in usage were seen, with average weekly consultation numbers ranging from 2 to 79 per hot hub (March to July 2020).

### 2. PHE FOI response

PHE data received detailed regional outbreaks or clusters of acute respiratory infections with possible/confirmed COVID-19 cases linked to GP surgeries. Table 1 shows this data as monthly totals from March to December 2020, alongside numbers of English face-to-face GP consultations obtained from NHS Digital. ^8^ In the March-August timeframe covered by model usage in this study, 25 outbreaks or clusters with possible/confirmed COVID-19 cases were reported in England in the context of over 104 million face-to-face GP appointments. This represented less than 2% of all COVID-19 outbreaks or clusters in England [calculated using data in COVID-19 surveillance reports]. ^6, 7^ Numbers increased in October and November, alongside rising face-to-face appointment levels, but continued to represent a small proportion of totals. ^6, 7^ Final figures may in fact be lower after investigation of unconfirmed cases by local PHE Health Protection Teams.

**Table 1.** Possible COVID-19 outbreaks/clusters and total face-to-face consultations in English general practice surgeries, March-December 2020.

Month *	North East	Yorkshire and Humber	North West	East of England	East Midlands	West Midlands	South East	South West	London	England	Monthly GP face-to-face appointments, England **
**March**	0	0	0	0	0	0	0	0	0	**0**	31,841,558
**April**	0	0	0	0	0	0	1	1	0	**2**	14,960,202
**May**	0	0	1	5	0	0	0	0	1	**7**	15,459,694
**June**	0	3	4	0	2	0	2	0	2	**13**	19,526,124
**July**	0	1	0	0	0	1	0	0	1	**3**	22,352,108
**Aug**	0	2	2	0	0	0	0	2	1	**7**	20,763,100
**Sept**	3	1	2	0	2	2	1	0	3	**14**	30,185,304
**Oct**	1	9	25	5	8	23	7	18	3	**99**	33,741,240
**Nov**	10	8	9	10	12	24	13	3	13	**102**	27,773,964
**Total**	**14**	**24**	**43**	**20**	**24**	**50**	**24**	**24**	**24**	**247**	216,603,294

* Monthly data calculated from weekly totals given in Public Health England response to FOI request. [March: 02/03/2020 to 29/03/2020; April: 30/03/2020 to 26/04/2020; May: 27/04/2020 to 31/05/2020; June: 01/06/2020 to 28/06/2020; July: 29/06/2020 to 26/07/2020; Aug: 27/07/2020 to 30/08/2020; Sept: 31/08/2020 to 27/09/2020; Oct: 28/09/2020 to 01/11/2020; Nov: 02/11/2020 to 29/11/2020]. Some 'possible' COVID-19 cases reported may be excluded after investigation by local PHE Health Protection Teams, and final numbers may therefore be lower.

** Face-to-face general practice appointment data obtained from NHS Digital. ^8^

## Discussion

### Model selection

All CCGs reported the use of zoned practices, hubs and/or home visits in varying combinations for face-to-face GP consultations. Factors influencing model selection included COVID-19 appointment demand, presence of pre-established collaborative networks and GP preferences to provide continuity of care. On-going model assessments enabled responsiveness to changing demand, mainly through altered hub availability, but also by changed provision of home monitoring for COVID-19 patients.

CCG mergers on 1st April 2020, which decreased total numbers from 191 to 135, may also have impacted model patterning within CCGs. While the ‘hot hubs + cold practices’ model combination #2 was the most frequently used nationwide, larger CCGs tended to report ‘mixed’ models, giving model combination #5 the greatest overall coverage ( Figure 2). Patterning within these mixed model combinations (#5-#7) could not always be identified, but in one CCG five areas of zoning and a sixth using a hot hub were identified, and in some others more diffuse patterning was noted.

### Variations within models

The distinction between the zoned practice and hub models used is not as clear as indicated in our *Introduction.* The co-location of hot hubs with cold services means that the requirement for strict management between hot and cold areas is as important here as in the zoned practice model. Indeed, several CCGs using co-located hubs and some using zoned practices specified the use of separate entrances and exits, with some also indicating separate car parking facilities. By contrast, other zoned practices shared more similarities with distinctly sited hubs, where red and green areas were split between main and branch surgeries for example, or additional structures such as portacabins were used on site, to separate patient cohorts registered within one practice. Thus, it is not necessarily the case that the hub model provides clearer separation and thereby simpler IPC adherence than zoned practices, as indicated in the guidelines. ^2, 4^

Usage of home visits also varied. In at least two CCGs this was the main or only form of COVID-19 face-to-face consultation. In 30% of CCGs however, the use of specific hot and/or cold home visits was not reported. Home monitoring, via pulse oximetry for example, was an additional service indicated by a small number of CCGs at the time of reporting.

### SARS-CoV-2 transmission

While the risk of transmission can be minimised in face-to-face consultations, it cannot be eliminated, particularly with the significant pre-symptomatic and asymptomatic transmission associated with COVID-19. ^9^ PHE data shows a total of 247 possible or confirmed COVID-19 outbreaks or clusters in English GP surgeries over the first nine months of the pandemic, in the context of more than 216 million face-to-face appointments. While these numbers are a small proportion of total (all source) outbreaks in the UK, investigation of any confirmed outbreak may guide future measures to reduce transmission.

The variability in possible/confirmed outbreaks or clusters in general practice over time, as well as by region (shown in Table 1), may partly be accounted for by changing COVID-19 incidence rates, but also by other factors including differences in testing rates, population risk factors, and the numbers and means of delivery of face-to-face consultations taking place locally.

### Evaluation

In addition to minimising SARS-CoV-2 transmission, the success of IPC delivery models used during the pandemic will be measured in terms of the management of COVID-19 and other conditions, and the perspectives of patients and staff. Several patient surveys have now been undertaken. ^10^^–^^12^ The authors have also reported on a survey of the public, investigating experiences and understanding of the changes to general practice. ^15^ These patient findings, taken together with staff perspectives ^3^ and quantitative evaluations should provide helpful insights into the effective use of the adapted models.

### Limitations

There are several limitations to this study. As already described, terms used to describe novel concepts (e.g., hot/cold hubs, zoning) varied and could not readily be checked. This may, for a small number of CCGs, have led to misinterpretation of responses and consequent misassignment to model combinations. Studies at Primary Care Network or practice level would likely reveal more granular detail, particularly with respect to the ‘mixed model’ combinations identified. In terms of the PHE FOI request, data on outbreaks/clusters from ‘GP surgeries’ was provided, and it is unclear whether this includes hubs and all home visiting services. Finally, the service delivery details provided by CCGs were mainly received in summer 2020 and, as has been identified, these are dynamic models and may since have been modified.

## Conclusions

This study provides an overview of nationwide IPC adaptation of face-to-face GP consultations in the spring and summer of 2020, with varied and dynamic models implemented to suit different and changing local conditions. Numbers of COVID-19 outbreaks linked to English GP surgeries indicate their relative success in minimising transmission. Shared learning from evaluation of these outbreaks across the different models may be instructive however for future management of SARS-CoV-2 transmission within general practice, where numbers of face-to-face consultations will need to increase again in future. Broader evaluation of the changes is also needed, including analysis of their impact on the management of non-COVID conditions, as well as on staff and patients. Taken together, this will identify beneficial elements of the rapidly enforced adaptations to inform practice both during this pandemic and beyond.

## Data Availability

The Re-use of Public Sector Information Regulations (RPSI) 2005 and copyright requirements have been invoked as imposing requirements around certain types of further use of survey data provided by some Clinical Commissioning Groups (CCGs). This may also apply to data received from other CCGs and Public Health England (PHE) and it is therefore not possible to share this data under the terms of the Creative Commons Attribution 4.0 International license (CC-BY 4.0). The data may be available from individual CCGs and PHE on request, with reference to the authors and this publication. Alternatively, Freedom of Information requests similar to those made by the authors may be used. Full details of these are provided in the *Extended data* and *Methods* section. Figshare: Survey sent to Clinical Commissioning Groups in England, https://doi.org/10.6084/m9.figshare.14156741.v1 ^13^ Figshare: Questions sent to Public Health England, https://doi.org/10.6084/m9.figshare.14156762.v1 ^14^ Data are available under the terms of the Creative Commons Attribution 4.0 International license (CC-BY 4.0).
