# Modifications to the delivery of NHS face-to-face general practice consultations during the COVID-19 pandemic in England

**DOI:** 10.12688/f1000research.52161.2

**Published:** 2021-08-20

**Authors:** Lorna J. Duncan, Kelly F.D. Cheng

**Affiliations:** 1 Centre for Academic Primary Care, Population Health Sciences, Bristol Medical School, University of Bristol, Bristol, UK; 2 Bristol Medical School, University of Bristol, Bristol, UK

**Keywords:** COVID-19, SARS-CoV-2, coronavirus, general practice, primary care, face-to-face consultation, delivery model, transmission

## Abstract

**Background: **To minimise transmission of SARS-CoV-2, the virus causing COVID-19, delivery of English general practice consultations was modified in March 2020 to enable separation of diagnosed or suspected COVID-19 patients from others. Remote triage and consultations became the default, with adapted face-to-face contact used only when clinically necessary. This study aimed to identify the modified face-to-face delivery models used nationwide in spring/summer 2020. Information was also sought concerning COVID-19 outbreaks linked to English general practice.

**Methods: **In June 2020, a survey was sent by email to the 135 Clinical Commissioning Groups (CCGs) in England to identify local organisation of face-to-face general practice consultations since March 2020. An email was  sent to Public Health England (PHE) requesting data on COVID-19 outbreaks linked to general practice.

**Results: **All CCGs responded. Between March and July 2020, separation of COVID-19 patients from others was achieved using combinations of the following models:

1. zoned surgeries (reported by 47% of CCGs), where COVID-19 and other patients were separated within their own practice;
2. ‘hot’ or ‘cold’ hubs (reported by 90% of CCGs), separate sites where COVID-19 or other patients registered at one of several collaborating practices were seen;
3. ‘hot’ and ‘cold’ home visits (reported by 70% of CCGs).

One of seven combinations of these models was used across each CCG, with some flexibility shown according to changing demand through hub availability.

PHE data indicated 25 possible or confirmed COVID-19 outbreaks or clusters in English general practice to July 31st 2020.

**Conclusions: **Varied, flexible ways of delivering face-to-face general practice consultations were identified.  Analysis of the modified delivery in terms of management of COVID-19 and other conditions, and impacts on staff and patients, together with learning from investigations into confirmed COVID-19 outbreaks, may both aid future pandemic management and identify beneficial elements for practice beyond this.

## Introduction

 In March 2020 it was estimated that more than 80% of patients with COVID-19 would not require hospitalisation. ^1^ It was likely therefore that many would seek treatment in general practice. In order to minimise transmission of the causative severe acute respiratory syndrome coronavirus 2 (SARS-CoV-2) during general practice (GP) consultations, NHS England’s Standard Operating Procedure was revised in March 2020 to a remote triage and consultation default, with adapted models for face-to-face contact used only when clinically necessary. ^2^ The use of telephone, video and online consultations in English general practice has been studied elsewhere. ^3^ In this paper we report on the delivery of face-to-face general practice consultations during the first wave of the pandemic, in spring/ summer 2020.

The requirement to separate patients with diagnosed or suspected COVID-19 [‘COVID-19’ patients] from others during clinically necessary face-to-face consultations was evident. NHS guidance suggested three possible ways to manage patients, premises and workforce for optimal Infection Prevention and Control (IPC): ^2^

(i) Zoned practices: In this model, patient cohorts would be separated within their own practices. Designated areas e.g., ‘red’ and ‘green’ zones, would be used to manage COVID-19 and other patients, respectively. Careful management would be needed to minimise cross-contamination between groups, including separate walkways and consultation rooms, and staff allocated to one zone only. Zoning could therefore be impractical in some surgeries.
(ii) Hot and cold hubs: A general practice hub would be designated as either ‘hot’ or ‘cold’, to treat COVID-19 or other patients respectively. It would be available to patients registered at one of several locally collaborating practices. With hot hubs sited separately to non-COVID services, IPC procedures could be more straightforward than in zoned practices. ^2, 4^
(iii) Dedicated home visiting: Home visiting services, modified to minimise cross-contamination, would be necessary for patients unable to access other face-to-face services, or where such provision was otherwise considered appropriate during the pandemic. Staff would work exclusively with COVID-19 or other patients, and work undertaken during visits would be maximised to limit additional face-to-face consultations. This service could be organised collaboratively, such as across Primary Care Networks, or by individual practices.

NHS guidance indicated decisions regarding model use were to be determined locally, in agreement with the relevant Clinical Commissioning Group (CCG) responsible for planning and commissioning NHS health care services in the area. It also recognised that flexible models could be required as patient demand and workforce capacity fluctuated throughout the pandemic. ^4^

While risk of transmission could be minimised with these measures however, it could not be eliminated in any face-to-face setting, particularly with the significant pre-symptomatic and asymptomatic transmission now known to be associated with COVID-19. ^5^ Indeed, it was postulated that hot hubs could become ‘lightning rods’ for transmission without strict adherence to IPC guidelines ^6^; and any weakness in the isolation of hot and cold zones would also increase potential for transmission. ^7^ In addition, the shortage of appropriate personal protective equipment such as masks and gloves at that time clearly increased risk in all face-to-face work. ^8^ As part of Public Health England’s pandemic surveillance, COVID-19 outbreaks were monitored for links to various settings. Numbers of incidents from hospitals, care homes, educational settings, prisons, workplaces and food outlets were published in weekly COVID-19 surveillance reports. ^9^^,^^10^ Data for general practice was not detailed however.

This study aimed to identify the ways in which local delivery of NHS face-to-face general practice was re-organised across England during the first wave of the pandemic, and to obtain available information on COVID-19 outbreaks linked to this setting. 

## Methods

### 1. CCG survey

A cross-sectional survey of the 135 CCGs in England was conducted to identify how face-to-face general practice consultations were delivered nationwide in spring/summer 2020. 

*Survey design*

Survey questions were devised by the study team. They concerned models of face-to-face consultations used and the patient populations each were available to; prior use of the hub model; and planned evaluations. Questions were pre-tested with a researcher experienced in survey design, two CCGs and one provider of primary healthcare. Minor changes to wording were made for clarity. The final questionnaire is available as *Extended data.* ^11^

*Data collection*

Questions were sent by email to all CCGs in June 2020 under the Freedom of Information (FOI) Act 2000. This legislation enables public access to recorded information held by public authorities in England. Responsibility for cleansing data lies with the authorities responding to FOI requests ^12^ and research ethics approval was not required.

Individual CCGs were identified on the NHS England website and their FOI procedures followed. FOI regulations mandate a response timeframe of 20 working days. Where, rarely, replies were not received within 25 working days, follow-up emails were sent, and telephone calls made if necessary. 

*Data analysis*

Full responses were collated in an Excel spreadsheet for analysis. Additional columns were used to summarise use of hot hubs, cold hubs, zoned practices and home visits for each CCG. Queries regarding response interpretation were discussed during regular online meetings (June-October) and in on-going email contact between the authors. Internet searches and occasional email / telephone communication with CCGs were also used for clarification (e.g. of whether specified ‘hot sites’ were hubs or zoned practices) or updates.

Emerging themes were discussed by the authors and further columns added to the spreadsheet indicating flexibility in operational hub numbers and co-location of hot hubs with cold services. Data was analysed individually and jointly in an iterative process as further responses were received.

With the identification of distinct patterns of face-to-face delivery, each CCG was assigned to a model combination of best fit for comparison.

### 2. Public Health England (PHE) query

An FOI request was emailed to PHE in December 2020 requesting information on COVID-19 outbreaks linked to general practice similar to that given for other settings in the weekly COVID-19 surveillance reports ^9^^,^^10^ (questions asked are available as *Extended data* ^13^). Additional emails and telephone calls with PHE and NHS England & NHS Improvement were used for clarification between February and April 2021.

## Results

### 1. CCG survey

*Responses*

Replies were received from all CCGs, 99% by July 2020, with the final response received on 2 ^nd^ October.

*Response interpretation*

Terminology used in responses varied – ‘hot sites’, and ‘resilience hubs’ could refer to the same or different services for example, as could ‘green’ and ‘amber’ colour coding. Provision for non-COVID patients was also sometimes unclear. Complete response sets (including any documentation provided on model pathways and usage data), were therefore used, together with internet searches and further CCG contacts, to interpret and categorise all face-to-face consultation types according to the models in this report.

The following interpretation of the data is the authors’ own and has not been approved or otherwise by the CCGs. It relates to the period between March 2020 and CCG response dates, largely June/July 2020. A summary spreadsheet supporting these findings, is available as *Underlying data*. ^14^

*Adapted delivery models*

**Figure 1.**
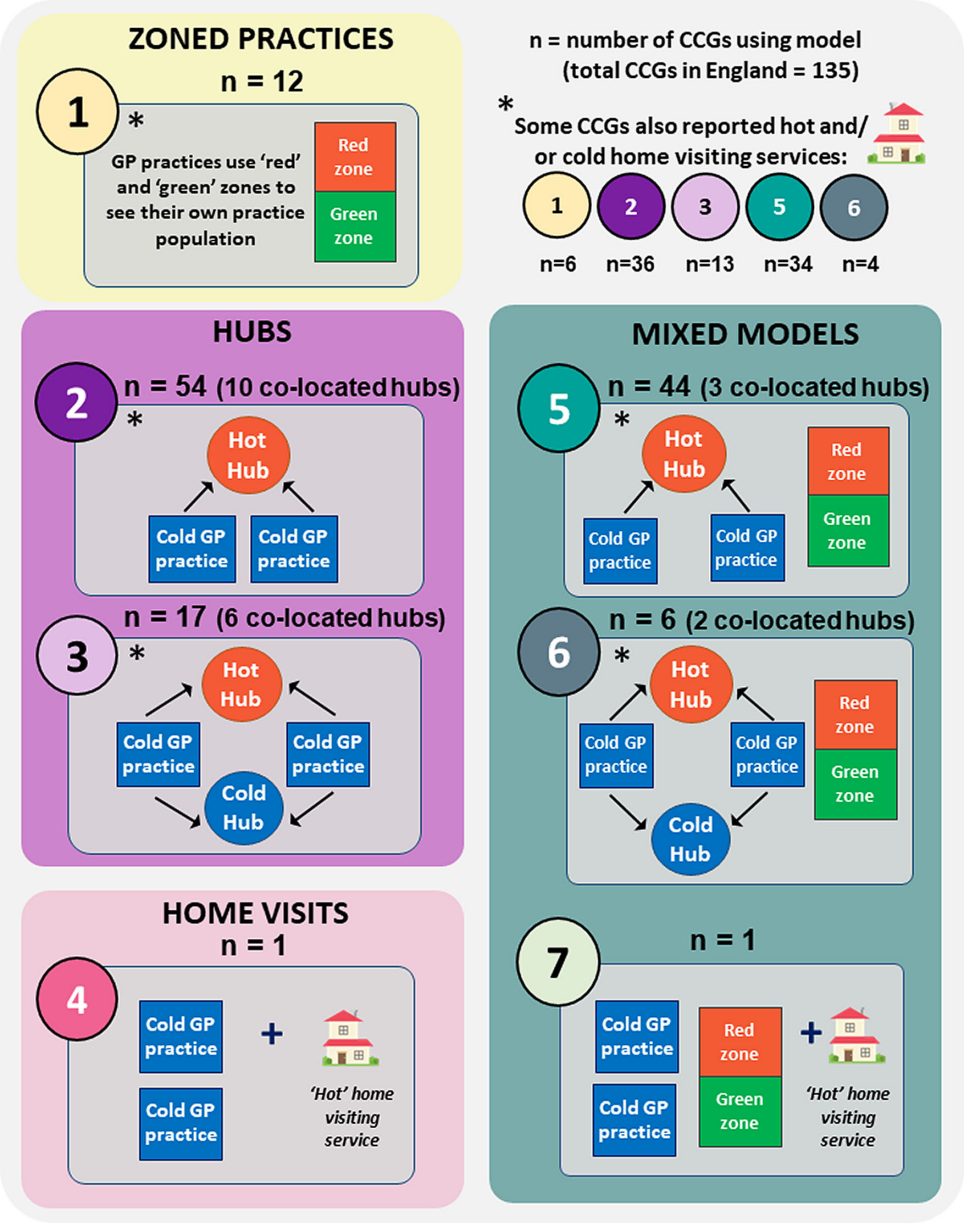
Models used to separate COVID-19 and other patients during face-to-face NHS GP consultations in England. Authors’ interpretations of CCG responses according to the following definitions:
•Zoned practice: co-location of hot and cold services on a single site, serving own practice list•Hot or cold hub: site of multi-practice working for COVID-19 or other patients respectively

General practice face-to-face delivery was modified in each CCG using combinations of the three indicated models:

(i) Zoned practices (model 1, Figure 1), available to the entire patient populations served, were reported by 47% of CCGs. All but 12 of these also used hubs at the time of reporting. Most commonly, two closed ‘red’ and ‘green’ areas with different entrances and exits were used. Rarely, cohorts were separated temporally, with COVID-19 patients alone seen at specific times. This model was described in updated NHS England guidance (version 2, dated 5 ^th^ April 2020) for surgeries where provision of separate spaces was not possible. ^2^
(ii) ‘Hot’ or ‘cold’ hubs (models 2 and 3, Figure 1), were reported by 90% of CCGs. All of these had hot hubs, with 23% also using cold hubs. Hubs were generally available to the entire combined patient populations served. Occasionally however, cold hubs had more specific uses - a ‘super-green’ hub for example, for patients requiring additional shielding, and a ‘purple’ hub for routine treatments such as vaccinations and maternity checks. Hub reach extended from several practices to entire CCGs and, in two instances, access was shared across neighbouring CCG boundaries. Hubs were sited in re-purposed buildings (surgeries for example, or hubs usually offering extended GP access), a racecourse and temporary structures (e.g., portacabins and marquees), or provided as drive-through facilities. Most CCGs reported using hubs prior to the pandemic, mainly for the provision of extended hours GP access.
  The use of ‘co-located’ hubs was indicated in 21 CCGs, whereby hot hubs were sited adjacent to cold hubs (n = 4) or cold practices (n = 17).
(iii) ‘Hot’ and/or ‘cold’ home visiting services, were reported by 70% of CCGs (24% of these detailed COVID-19 appointments only, 31 specified hot and cold visits and the remaining 39 CCGs did not indicate COVID-19 status). While these generally served patients unable to access other face-to-face services, they were the main form of face-to-face provision for COVID-19 patients in two CCGs. Delivery could be provided by practices, collaborative networks or CCG acute visiting services, and in some cases operated out of hubs. Home monitoring of COVID-19 patients, via delivery of pulse oximeters was also available in nine CCGs, while two provided transport to face-to-face sites.

The different means of delivering face-to-face services were compared for each CCG. Overall approaches taken were found to fit one of seven model combinations, albeit with some distinctions, notably different usage of home visits and co-located hubs.

**Figure 2.**
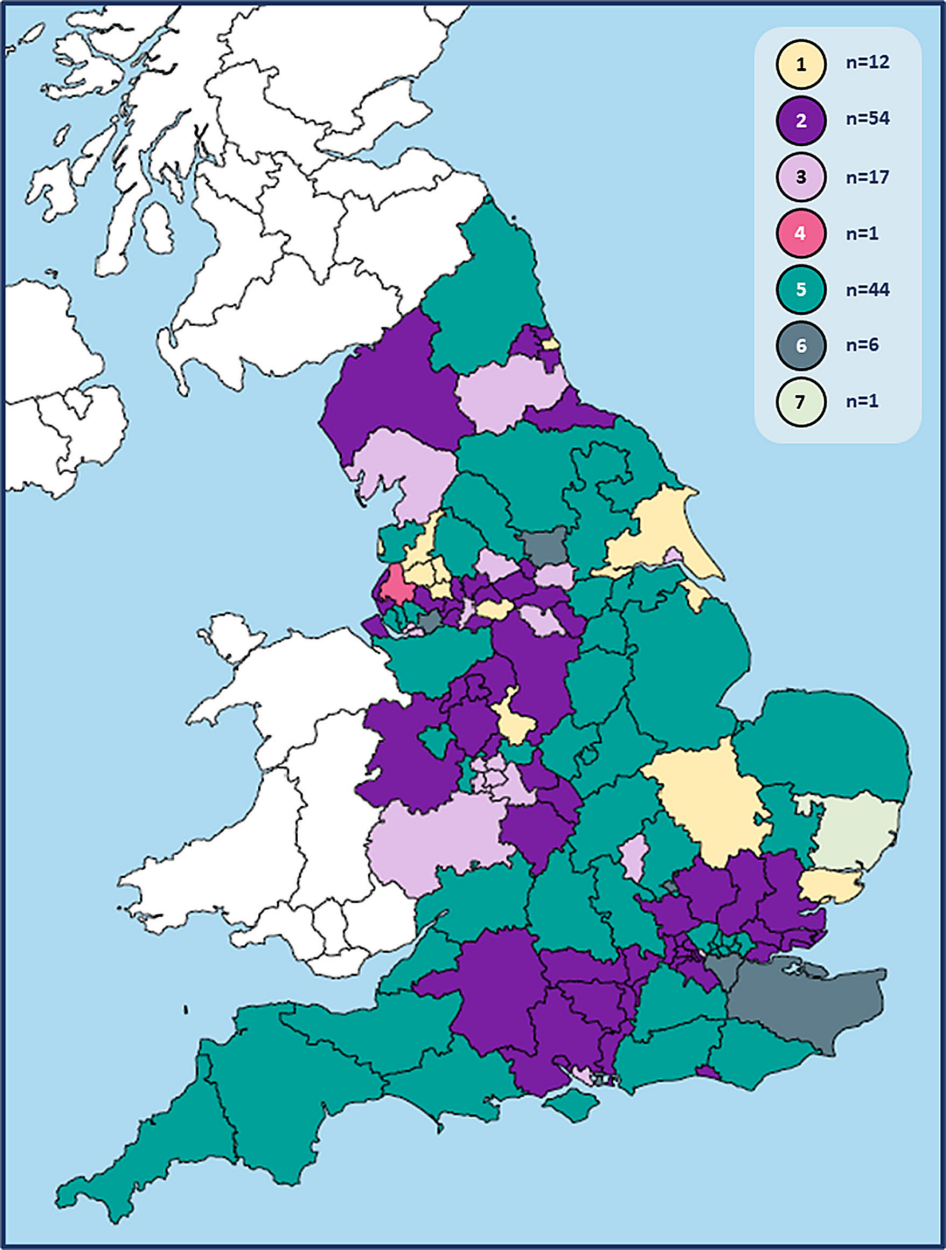
Local models used to separate COVID-19 and other patients during face-to-face GP consultations across England. Model combinations:* 1: zoned practices (+/- home visits) 2: hot hubs + cold practices (+/- home visits) 3: hot hubs, cold hubs + cold practices (+/- home visits) 4: hot home visits + cold practices 5: hot hubs, cold hubs + zoned practices (+/- home visits) 6: hot hubs, cold hubs, zoned practices + cold practices (+/- home visits) 7: zoned practices, cold practices + hot home visits. *Authors’ interpretations of CCG responses according to the following definitions:
•Zoned practice: co-location of hot and cold services on a single site, serving own practice list•Hot or cold hub: site of multi-practice working for COVID-19 or other patients respectively N.B. 16 CCGs did not describe face-to-face consultations for ‘cold’ patients. 15 of these were assigned to model combination 2 as hot hubs were described which were not co-located with cold services; and 1 was assigned to model combination 5. The face-to-face delivery data we have presented was correct between March 2020 and the date of response [by 30 ^th^ July (n = 134) and October (n = 1) 2020].

The seven model combinations are illustrated in Figure 1, with their distribution among CCGs shown in Figure 2. While the ‘hot hubs + cold practices’ model combination (#2) was the most frequently used nationwide, larger CCGs tended to report ‘mixed’ models, giving model combination #5 (‘hot hubs + cold practices + zoned practices’) the greatest geographical coverage.

*Evaluations and flexible models*

87% of CCGs reported on-going, complete or intended reviews, generally of hub and/or telephone triage use, although one CCG was considering its drive-through model's potential for influenza vaccinations, and others were focusing on staff/ patient perspectives. 25 CCGs reported reviewing usage to facilitate dynamic models, with hubs either available but as yet unused (n = 3) or numbers being flexed up and/or down (n = 22). [Assignment to model combinations 1-7 was based on provision at time of reporting.] Indeed, four of the twelve CCGs assigned to ‘zoned practice’ model #1 reported having hot hubs available if needed. In mid-October, with COVID-19 incidence rising in the second wave of the pandemic, contact with three of these, each in areas of mandated local lockdowns, ^9, 10^ revealed that, while hubs remained available, escalation plans had not yet been necessary. Some other CCGs also indicated that only some of their potential hubs had been required.

17 CCGs provided data on face-to-face contact across 21 hot hubs. While representing only a small proportion of total hubs, wide variations in usage were seen, with average weekly consultation numbers ranging from 2 to 79 per hot hub (March to July 2020).

### 2. PHE query 

PHE supplied regional data on confirmed and suspected COVID-19 outbreaks or clusters linked to GP surgeries in their response. More specific detail could not be provided due to potential deductive disclosure. Table 1 shows this data as monthly totals from March to December 2020, alongside numbers of GP appointments (including face-to-face, telephone and online consultations and home visits) obtained from NHS Digital data. ^15^ During the March-August timeframe of this study, 25 outbreaks or clusters were reported in English general practice. This represented less than 2% of outbreaks in all settings [using data in COVID-19 surveillance reports] in the context of over 99 million general practice appointments (53% of these were for face-to-face consultations). ^9, 10^ Final figures may in fact be lower after investigation of unconfirmed cases. Numbers of outbreaks in general practice rose in October and November, alongside increases in both COVID-19 incidence and appointments. Comparison with ‘all settings’ outbreak totals was not possible due to changes in reporting (from English to UK-wide data). ^9, 10^

Additional communication with staff at PHE and NHS England & NHS Improvement in one region, revealed that transmission between staff was much more common there than that between staff and patients in general practice outbreaks.

**Table 1.** Confirmed and suspected COVID-19 outbreaks/ clusters and appointment numbers in English general practice, March-December 2020.

Month *	North East	Yorkshire and Humber	North West	East of England	East Midlands	West Midlands	South East	South West	London	England	Monthly GP appointments, England **
**March**	0	0	0	0	0	0	0	0	0	**0**	23,771,858
**April**	0	0	0	0	0	0	1	1	0	**2**	15,835,467
**May**	0	0	1	5	0	0	0	0	1	**7**	16,375,240
**June**	0	3	4	0	2	0	2	0	2	**13**	20,640,372
**July**	0	1	0	0	0	1	0	0	1	**3**	22,413,336
**Aug**	0	2	2	0	0	0	0	2	1	**7**	20,083,871
**Sept**	3	1	2	0	2	2	1	0	3	**14**	26,655,638
**Oct**	1	9	25	5	8	23	7	18	3	**99**	28,236,193
**Nov**	10	8	9	10	12	24	13	3	13	**102**	24,999,273
**Total**	**14**	**24**	**43**	**20**	**24**	**50**	**24**	**24**	**24**	**247**	199,011,248

* Monthly data calculated from weekly totals given in Public Health England response to FOI request. [March: 02/03/2020 to 29/03/2020; April: 30/03/2020 to 26/04/2020; May: 27/04/2020 to 31/05/2020; June: 01/06/2020 to 28/06/2020; July: 29/06/2020 to 26/07/2020; Aug: 27/07/2020 to 30/08/2020; Sept: 31/08/2020 to 27/09/2020; Oct: 28/09/2020 to 01/11/2020; Nov: 02/11/2020 to 29/11/2020]. Some 'possible' COVID-19 cases reported may be excluded after investigation by local PHE Health Protection Teams, and final numbers may therefore be lower.

** General practice appointment data (for face-to-face, telephone and online consultations and home visits) obtained from NHS Digital. ^15^

## Discussion

### Model use 

All CCGs reported using zoned practices, hubs and/or home visits in various combinations. Factors influencing selection included appointment demand, existence of collaborative networks, site adaptability and preferences for providing care continuity. On-going assessment enabled responsiveness to changing demand, mainly through altered hub availability.

50 CCGs were assigned to ‘mixed models’ using both hubs and zoned practices (combinations #5 and #6). This was in part related to the scheduled CCG mergers which took place on 1 ^st^ April 2020 - one week into the first national lockdown - decreasing total numbers from 191 to 135. Thirteen of the eighteen emergent CCGs were assigned to mixed model combinations, and two of these reported distinct model usage aligned with their component former CCGs. It is possible that more detailed study of others would reveal similar patterns. Meanwhile, where model patterning could be identified in the 37 ‘mixed models’ CCGs not involved in mergers, either CCG-wide patterns or distinct areas of zoning and hubs were revealed.

### Variations within models

The distinction between zoned practice and hub models used was not as clear as indicated *.* Where hot hubs were co-located with cold services the requirement for strict management between hot and cold areas was as important as in zoned practices. Indeed, several CCGs using co-located hubs or zoned practices specified use of separate entrances and exits, with some also reporting separate parking facilities. Other zoned practices meanwhile shared more similarities with distantly sited hubs, where red and green areas were split between main and branch surgeries for example, or where additional structures such as portacabins were used, to separate patient cohorts. Thus, it was not the case that the hub model always provided clearer separation and thereby simpler IPC adherence than zoned practices, as indicated in the guidelines. ^2, 4^

Use of dedicated home visits also varied. While in at least two CCGs this was the main or only form of COVID-19 face-to-face consultation, the use of specific hot and/or cold home visits was not reported by 30% of CCGs. This may be due to the service being operated outside primary care, as reported by some. Home monitoring via pulse oximetry was also offered by a small number of CCGs with, in one case, trained volunteers delivering the necessary equipment.

Further adaptations were shown by the temporary use of alternative non-healthcare settings and car-based models.

### SARS-CoV-2 transmission

The 25 COVID-19 outbreaks or clusters linked to general practice during the 4-month study period equated to less than 2% of all-settings totals. Each setting type however has unique characteristics impacting transmission, making specific comparison between them problematic. ^10^ In General Practice for example, while staff may remain on site for long periods, patient consultations average around 10 minutes’ duration and IPC measures, including remote triage, may have further reduced this. ^16^ Furthermore, the facility to see COVID-19 patients at sites located completely separately from other patients is not available in all healthcare settings. Conversely, turnover of those on site may be relatively high and other factors such as the vulnerability of patients to COVID-19, may also impact.

In one English region, investigations showed there were considerably more outbreaks between staff in general practice than between patients and staff and factors indicated above may have contributed to this. It was not possible to ascertain whether this was a common feature of transmission in general practice nationally. If this were to be the case however, not only may shared learning from investigations assist in reducing such incidents, it may also help to allay the safety concerns of patients who remain wary of contacting general practice, identified in our linked survey and those of others. ^17^^-^^19^

Just as differences between settings impact transmission, changes in numbers of outbreaks in general practice over time, as well as by region (see Table 1), reflect multi-factorial influences and comparison may be misleading. ^10^ The changes seen could have been partly accounted for by differing COVID-19 incidence rates, but also by other factors including differences in testing rates, population risk factors, and the numbers and types of interactions (consultations, staff interactions, visitors to site, etc.) taking place.

### Strengths and limitations

The study methodology and data received have both strengths and limitations. The use of a national survey, CCG level data collection and FOI requests will be considered in turn.

While this survey has provided a national picture of face-to-face general practice delivery in the first months of the pandemic in England, it no longer reflects current practice. It nevertheless enables review of early adaptations with the benefit of increased understanding of SARS-CoV-2 transmission, and of various impacts of the modified models on staff and patients. ^5, 17- 19^ It may be used to inform case site selection for more in-depth analysis to clarify issues such as those raised in this report, and to plan responses to further rises in incidence and new epidemics/pandemics.

The use of CCG level data facilitated a manageable national overview in an initial 6-week study. It is likely however that questioning at practice level would have revealed greater nuance - in terms of model selection for example, practice size, staffing, local population and geographical factors may have been revealed to impact this, in addition to those indicated in our CCG responses. Some CCG boundaries were also revealed to be somewhat flexible in terms of service delivery, with a degree of hub sharing reported. Close working was further indicated in some responses which were completed by one CCG on behalf of up to five others. The 2020 CCG mergers have already been discussed, and another round in April 2021 has since reduced numbers to 106. With all CCGs scheduled to merge across larger Integrated Care System boundaries in April 2022, a degree of complexity will be added to any future study utilising the current findings.

FOI requests, identified as a preferred data collection method by CCGs, ^20^ ensure high response rates within a mandated 4-week timeframe. They do not however readily permit further questioning, and both questions and responses may therefore be open to misinterpretation. While further investigation was used to minimize error resulting from missing or ambiguous data, it is possible that models were misassigned in a small number of cases. [Data queries are noted in column J of the summary spreadsheet.] ^14^ An additional issue resulted from generic text within some responses requiring a further request to publish. While many CCGs agreed to this, different stipulations by others could not be met under the terms of the Open Access licence used and the data could not be published here.

PHE’s FOI response yielded data not publicly available indicating apparently low numbers of COVID-19 outbreaks linked to general practice. Confirmation of transmission in non-residential settings such as this is complicated however, with the potential of exposure to SARS-CoV-2 also occurring outside general practice. The specific context in which transmission took place (e.g. hot/cold site, activities undertaken on site, staff/ patient/ visitors affected) could not be identified from the data received.

## Conclusions

This study has provided an overview of adaptations of face-to-face GP consultations during the first four months of the COVID-19 pandemic in England, with varied and dynamic models implemented to suit different and changing local conditions. 25 COVID-19 outbreaks linked to general practice were reported nationally in this period. Shared learning from outbreak investigations may be instructive for future management of transmission; and evaluation of the delivery modifications described, including analysis of impacts on the management of non-COVID conditions, and on staff and patients, may also be used to identify beneficial elements of the rapidly enforced adaptations to inform practice both during the COVID-19 pandemic and beyond.

## Data Availability

The Re-use of Public Sector Information Regulations (RPSI) 2005 and copyright requirements have been invoked as imposing requirements around certain types of further use of survey data provided by some Clinical Commissioning Groups (CCGs). This may also apply to data received from other CCGs and Public Health England (PHE) and it is therefore not possible to share this data under the terms of the Creative Commons Attribution 4.0 International license (CC-BY 4.0). The data may be available from individual CCGs and PHE on request, with reference to the authors and this publication. Alternatively, Freedom of Information requests similar to those made by the authors may be used. Full details of these are provided in the *Extended data* and *Methods* section. A summary spreadsheet of the authors' analysis of this data is also available: Figshare: Summary analysis of NHS face-to-face general practice models during the first wave of the COVID-19 pandemic in England March to July 2020, https://doi.org/10.6084/m9.figshare.14852517.v1. ^14^ Data are available under the terms of the Creative Commons Attribution 4.0 International license (CC-BY 4.0). Figshare: Survey sent to Clinical Commissioning Groups in England, https://doi.org/10.6084/m9.figshare.14156741.v1. ^11^ Figshare: Questions sent to Public Health England, https://doi.org/10.6084/m9.figshare.14156762.v1. ^13^ Data are available under the terms of the Creative Commons Attribution 4.0 International license (CC-BY 4.0).
