# Modifications to the delivery of NHS face-to-face general practice consultations during the COVID-19 pandemic in England

**DOI:** 10.12688/f1000research.52161.3

**Published:** 2021-11-01

**Authors:** Lorna J. Duncan, Kelly F.D. Cheng

**Affiliations:** 1 Centre for Academic Primary Care, Population Health Sciences, Bristol Medical School, University of Bristol, Bristol, UK; 2 Bristol Medical School, University of Bristol, Bristol, UK

**Keywords:** COVID-19, SARS-CoV-2, coronavirus, general practice, primary care, face-to-face consultation, delivery model, transmission

## Abstract

**Background: **In order to minimise transmission of SARS-CoV-2, the virus causing COVID-19, delivery of English general practice consultations was modified in March 2020 to enable the separation of patients with diagnosed or suspected COVID-19 from others. Remote triage and consultations became the default, with adapted face-to-face contact used only when clinically necessary. Face-to-face delivery modifications were decided locally and this study aimed to identify the different models used nationwide in spring/summer 2020.

**Methods: **In June 2020, a survey was sent by email to the 135 Clinical Commissioning Groups (CCGs) responsible for planning and commissioning NHS health care services in England to identify the local organisation of face-to-face general practice consultations since March 2020.

**Results: **All CCGs responded. Between March and July 2020, separation of patients with diagnosed or suspected COVID-19 (‘COVID-19 patients’) from others was achieved using the following models:

1. zoned practices (used within 47% of CCGs), where COVID-19 and other patients were separated within their own practice;
2. ‘hot’ or ‘cold’ hubs (used within 90% of CCGs), separate sites where COVID-19 or other patients registered at one of several collaborating practices were seen;
3. ‘hot’ and ‘cold’ home visits (used within 70% of CCGs).

For around half of CCGs, either all their GP practices used zoning, or all used hubs; in other CCGs, both models were used. Demand-led hub availability offered flexibility in some areas. Home visits were mainly used supplementally for patients unable to access other services, but in two CCGs, they were the main/only form of COVID-19 provision.

**Conclusions: **Varied, flexible ways of delivering face-to-face general practice consultations were identified.  Analysis of the modified delivery in terms of management of COVID-19 and other conditions, and other impacts on staff and patients, may both aid future pandemic management and identify beneficial elements for practice beyond this.

## Introduction

In March 2020 it was estimated that more than 80% of patients with COVID-19 would not require hospitalisation, ^1^ and it was likely that many would seek treatment in general practice. In order to minimise transmission of the causative severe acute respiratory syndrome coronavirus 2 (SARS-CoV-2) during general practice (GP) consultations, NHS England’s Standard Operating Procedure was revised in March 2020 to a remote triage and consultation default, with adapted models for face-to-face contact used only when clinically necessary. ^2^ The use of telephone, video and online consultations in English general practice has been studied elsewhere. ^3^ In this paper we report on the delivery of face-to-face general practice consultations across England during the first wave of the pandemic, in spring/summer 2020.

The need to separate patients with diagnosed or suspected COVID-19 [‘COVID-19’ patients] from others to minimise cross-infection during clinically necessary face-to-face consultations was evident. NHS guidance suggested three possible ways to manage patients, premises and workforce for optimal Infection Prevention and Control (IPC): ^2^

(i) Zoned practices: In this model, patient cohorts would be separated within their own practices. Designated areas e.g., ‘red’ and ‘green’ zones, would be used to manage COVID-19 and other patients, respectively. Careful management would be needed to minimise cross-contamination between groups, including separate walkways and consultation rooms, and staff allocated to one zone only. Zoning could therefore be impractical in some surgeries.
(ii) Hot and cold hubs: A general practice hub would be designated as either ‘hot’ or ‘cold’, to treat COVID-19 or other patients respectively. It would be available to patients registered at one of several locally collaborating practices. With hot hubs sited separately to non-COVID services, IPC procedures could be more straightforward than in zoned practices. ^2, 4^
(iii) Dedicated home visiting: Home visiting services, modified to minimise cross-contamination, would be necessary for patients unable to access other face-to-face services, or where such provision was otherwise considered appropriate during the pandemic. Staff would work exclusively with COVID-19 or other patients, and work undertaken during visits would be maximised to limit additional face-to-face consultations. This service could be organised collaboratively, such as across Primary Care Networks (groups of local GP practices), or by individual practices.

NHS guidance indicated decisions regarding model use were to be determined locally, and that these could require flexibility as patient demand and workforce capacity fluctuated throughout the pandemic. All decisions were to be made in agreement with the relevant Clinical Commissioning Group (CCG), ^4^ the organisation responsible for planning and commissioning NHS health care services in the area. In June 2020, 135 CCGs and 6761 GP practices were in operation across England, with each practice forming part of their local CCG. ^5^

This study aimed to identify the ways in which local delivery of NHS face-to-face general practice was re-organised across England during the first wave of the pandemic.

## Methods

A cross-sectional survey of the 135 CCGs in England was conducted to identify how face-to-face general practice consultations were delivered nationwide in spring/summer 2020.

*Survey design*

Survey questions were devised by the study team. They concerned models of face-to-face consultations used and the patient populations each were available to; prior use of the hub model; and planned evaluations. Questions were pre-tested with a researcher experienced in survey design, two CCGs and one provider of primary healthcare. Minor changes to wording were made for clarity. The final questionnaire is available as *Extended data.* ^6^

*Data collection*

Questions were sent by email to all CCGs in June 2020 under the Freedom of Information (FOI) Act 2000. This legislation enables public access to recorded information held by public authorities in England. Responsibility for cleansing data lies with the authorities responding to FOI requests ^7^ and research ethics approval was not required.

Individual CCGs were identified on the NHS England website and their FOI procedures followed. FOI regulations mandate a response timeframe of 20 working days. Where, rarely, replies were not received within 25 working days, follow-up emails were sent, and telephone calls made if necessary. 

*Data analysis*

Full responses were collated in an Excel spreadsheet for analysis. Additional columns were created to summarise the use of hot hubs, cold hubs, zoned practices and home visits within each CCG. Queries regarding response interpretation were discussed during regular online meetings (June-October 2020) and in on-going email contact between the authors. Internet searches and occasional email / telephone communication with CCGs were also used for clarification (to establish whether specified ‘hot sites’ were hubs or zoned practices for example) or updates.

Emerging themes were discussed by the authors and further columns added to the spreadsheet indicating flexibility in operational hub numbers and co-location of hot hubs with cold services. Data was analysed individually and jointly in an iterative process as further responses were received.

Common patterns of delivery across the 135 CCGs were then identified using the summary columns indicating services for COVID-19 patients (hot hubs, zoned practices and home visits). Initially, data for each CCG reporting the use of hot hubs was examined to check whether zoned practices and/or hot home visits were also being used in any of their GP practices. Consideration was then made of how other services (cold hubs, cold practices and/or cold home visits) were operating across these CCGs. A similar process was repeated for the remaining CCGs, in which zoned practices and/or hot home visits, but no hot hubs, had been reported.

In this way, a total of 12 different forms of model use were identified, each describing overall delivery of general practice for COVID-19 and non-COVID-19 patients across a CCG. Due to the varied reporting of home visiting services however (e.g., as hot and/or cold home visits, or with no COVID-19 specification provided, or with an indication that they were organised outside of general practice), it was considered that categorisation of CCGs on this basis could be misrepresentative. Those models which varied only in the reported use or otherwise of home visits were therefore merged, resulting in the amalgamation of 10 of the 12 models into 5. Together with the two remaining original models, in which hot home visits were specified as the only or major form of provision for COVID-19 patients, this yielded a final set of 7 models to which each CCG was assigned for comparison. Data on population density ^8^ and GP practice numbers ^5^ were also obtained and added to the summary spreadsheet to aid analysis.

## Results

*Responses*

Replies were received from all CCGs, 99% by 31 ^st^ July 2020, with the final response received on 2 ^nd^ October 2020.

*Response interpretation*

Terminology used in responses varied – ‘hot sites’, and ‘resilience hubs’ could refer to the same or different services for example, as could ‘green’ and ‘amber’ colour coding. Provision for non-COVID-19 patients was also sometimes unclear. Complete response sets (including any documentation provided on model pathways and usage data), were therefore used, together with internet searches and further CCG contacts, to interpret and categorise all face-to-face consultation types according to the models in this report.

The following interpretation of the data is the authors’ own and has not been approved or otherwise by the CCGs. It relates to the period between March 2020 and CCG response dates (largely June/July 2020), and reflects all models in use by GP practices within each CCG's boundaries. A summary spreadsheet supporting these findings, is available as *Underlying data.* ^9^

*Adapted delivery models*

**Figure 1.**
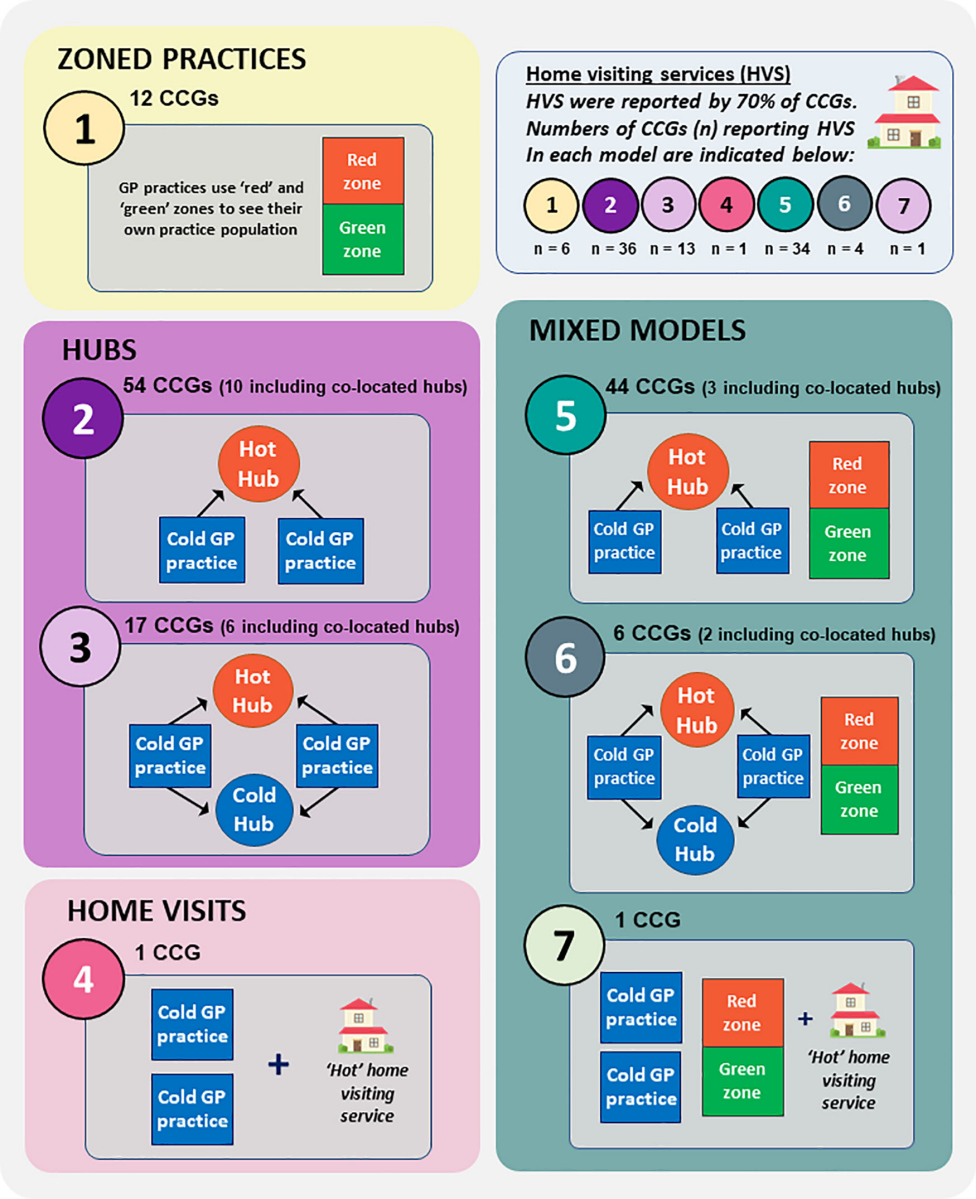
Models used to separate COVID-19 and other patients during face-to-face NHS GP consultations in England. Authors’ interpretations of CCG responses according to the following definitions:
•Zoned practice: co-location of hot and cold services on a single site, serving own practice list•Hot or cold hub: site of multi-practice working for COVID-19 or other patients respectively COVID-19 services shown as 'hot' or 'red'; non-COVID-19 services shown as 'cold' or 'green'. Models 1-4 are named according to their COVID-19 adaptation. Each CCG (n = 135) was assigned to one of the seven model combinations according to overall use by GP practices within its boundaries. Individual practices may have used one or more of the models indicated within combination models. Home visiting service use varied within models and is indicated separately in the top, pale blue box.

General practice face-to-face delivery was modified within each CCG using combinations of the three indicated models:

(i) Zoned practices (model 1, Figure 1), available to their entire patient populations, were reported as in use within 47% of CCGs. Most commonly, two closed ‘red’ and ‘green’ areas with different entrances and exits were used. Rarely, cohorts were separated temporally, with COVID-19 patients alone seen at specific times. This model was described in updated NHS England guidance (version 2, dated 5 ^th^ April 2020) for surgeries where provision of separate spaces was not possible. ^2^ 50 of the 63 CCGs in which zoned practices were operating indicated that hubs were also in use by some practices at the time of reporting.
(ii) ‘Hot’ or ‘cold’ hubs (models 2 and 3, Figure 1), were indicated as being used by some or all practices in 90% of CCGs. Each of these 121 CCGs had practices using hot hubs, with 23 also using cold hubs. Hubs were generally available to the entire patient populations of collaborating practices. Occasionally however, cold hubs had more specific uses - a ‘super-green’ hub for example, for patients requiring additional shielding, and a ‘purple’ hub for routine treatments such as vaccinations and maternity checks. Hub reach extended from several practices to entire CCGs and, in two instances, access was shared across neighbouring CCG boundaries. Hubs were sited in re-purposed buildings (surgeries for example, or hubs usually offering extended GP access), locations not previously used for healthcare including a racecourse and temporary structures (e.g., portacabins and marquees), or they were provided as drive-through facilities. Most CCGs reported use of hubs by their practices prior to the pandemic, mainly for the provision of extended hours GP access (after 6.30 pm Monday-Friday, and at weekends).
  The use of one or more ‘co-located’ hubs was indicated in 21 CCGs, whereby hot hubs were sited adjacent to cold hubs (4CCGs) or cold practices (17 CCGs).
(iii) ‘Hot’ and/or ‘cold’ home visiting services, were reported as available within 70% of CCGs (25 of these indicated they were for COVID-19 appointments only, 31 that specified both hot and cold visits were available, while the remaining 39 CCGs did not indicate COVID-19 status). While these generally served patients unable to access other face-to-face GP services, they were the main form of face-to-face GP provision for COVID-19 patients in two CCGs. Delivery could be provided by individual practices, collaborative networks or CCG acute visiting services, and in some cases operated out of hubs. Home monitoring of COVID-19 patients, via delivery of pulse oximeters was also reported by nine CCGs, while two provided transport to face-to-face sites.

The different means of delivering face-to-face services were compared for each CCG. Some reported a consistent approach to hot and cold service delivery by each GP practice. In other CCGs however, distinct local patterns of delivery were used. The combined approaches taken by all GP practices operating within each CCG were therefore assessed and all CCGs were found to fit one of seven forms of overall service delivery, albeit with some distinctions, notably the different use of home visits and of co-located hubs.

**Figure 2.**
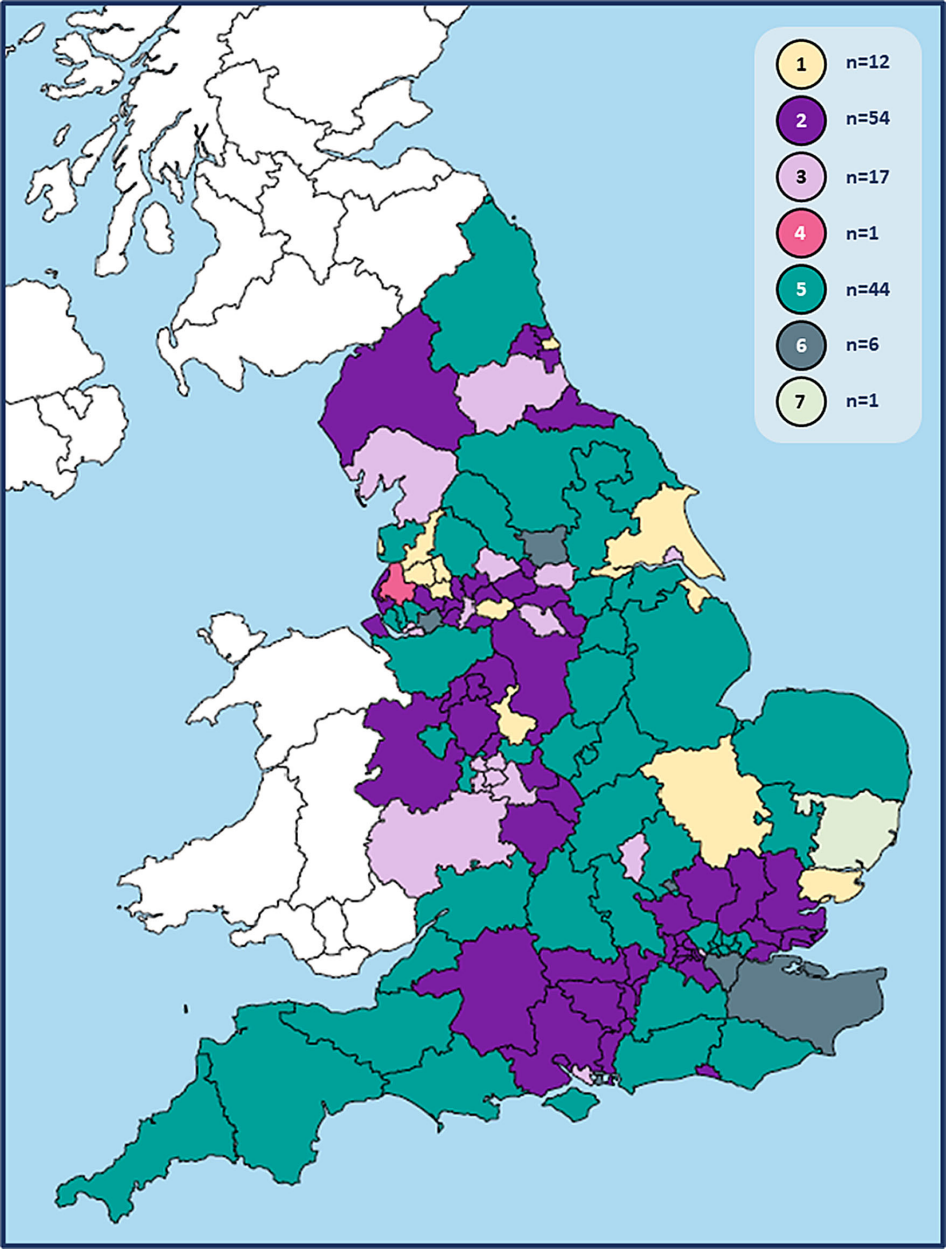
Local models used to separate COVID-19 and other patients during face-to-face GP consultations across England. Model combinations:* 1: zoned practices (+/- home visits) 2: hot hubs + cold practices (+/- home visits) 3: hot hubs, cold hubs + cold practices (+/- home visits) 4: hot home visits + cold practices 5: hot hubs, cold hubs + zoned practices (+/- home visits) 6: hot hubs, cold hubs, zoned practices + cold practices (+/- home visits) 7: zoned practices, cold practices + hot home visits. *Authors’ interpretations of CCG responses according to the following definitions:
•Zoned practice: co-location of hot and cold services on a single site, serving own practice list•Hot or cold hub: site of multi-practice working for COVID-19 or other patients respectively N.B. 16 CCGs did not describe face-to-face consultations for ‘cold’ patients. 15 of these were assigned to model combination 2 as hot hubs were described which were not co-located with cold services; and 1 was assigned to model combination 5. The face-to-face delivery data presented was correct between March 2020 and the date of response [by 30 ^th^ July (n = 134) and October (n = 1) 2020].

The seven model combinations and their use by CCGs are shown in Figure 1, and in the summary analysis table. ^9^ Their distribution across England is illustrated in Figure 2.

More than half of CCGs reported the use of a common model for COVID-19 patients by each of their GP practices (model numbers 1-4). Indeed, the ‘hot hubs + cold practices’ model combination (#2) was the most frequently used nationwide, employed by 40% of CCGs. Only 9% of CCGs used zoned practices alone, and 1% used home visiting services as their main or only form of provision for COVID-19 patients.

As Figure 2 illustrates however, geographically larger CCGs tended to report the use of ‘mixed’ models of delivery (numbers 5-7), giving model combination #5 (‘hot hubs + cold practices + zoned practices’) the greatest coverage across England. However, the physical size of a CCG did not necessarily reflect its population size with both remote rural areas and large conurbations contributing to the English landscape. Indeed, wide ranges in population density – from 63.8 to 16,427 people/km ^2^ – were seen in the different CCGs, and this range was similar for those assigned to the single (1-4) and mixed (5-7) COVID-19 models. ^8, 9^ Similarly, the numbers of GP practices in each CCG ranged from 7 to 214 in June 2020 and these too showed a broad spread among both single and mixed models. ^5, 9^

*Evaluations and flexible models*

87% of CCGs reported on-going, complete or intended reviews, generally of hub and/or telephone triage use, although one CCG was considering the potential of its drive-through model for influenza vaccinations, and others were focusing on staff or patient perspectives. 25 CCGs reported reviewing usage to facilitate dynamic models, with hubs either available but as yet unused (n = 3), or numbers being flexed up and/or down (n = 22). [Assignment to model combinations 1-7 was based on provision at time of reporting.] Indeed, four of the twelve CCGs assigned to the ‘zoned practice’ model #1 reported having hot hubs available if needed. In mid-October 2020, with COVID-19 incidence rising in the second wave of the pandemic, contact with three of these, each in areas with markedly higher case numbers than the national average and in mandated local lockdowns, ^10, 11^ revealed that, while their hubs remained available, escalation plans had not yet been necessary. Some other CCGs also indicated that only some of their potential hubs had been required.

17 CCGs provided data on face-to-face contact across 21 hot hubs. While representing only a small proportion of total hubs, wide variations in usage were seen, with average weekly consultation numbers ranging from 2 to 79 per hot hub (March to July 2020).

## Discussion

### Model use 

All CCGs reported the use of zoned practices, hubs and/or home visits in various combinations by their GP practices. 73% reported the use of hot hubs (but no cold hubs), either with or without zoned practices. A further 17% incorporated cold hubs into these models. Only 9% of CCGs used zoned practices alone and 1% used home visiting services as their major or only form of provision for COVID-19 patients. Factors influencing model selection included appointment demand, existence of local collaborative networks, adaptability of premises and preferences for providing continuity of care. Different workforce capacities are also likely to have influenced this, with almost 10% of GP practices in England run by single GPs, and 1 in 3 of these GPs estimated to be at high risk for COVID-19 infection. ^12^ On-going assessment in CCGs enabled responsiveness to changing demand, mainly through altered hub availability.

50 CCGs were assigned to ‘mixed models’ combinations, using both hubs and zoned practices within their boundaries (combinations #5 and #6). This was in part related to the scheduled CCG mergers which took place on 1 ^st^ April 2020 - one week into the first national lockdown - decreasing total numbers from 191 to 135. Thirteen of the eighteen emergent CCGs were assigned to mixed model combinations, and two of these reported distinct model usage aligned with their component former CCGs. It is possible that more detailed study of others would reveal similar patterns. Meanwhile, where model patterning could be identified in the 37 CCGs assigned to ‘mixed models’ combinations but not involved in mergers, either CCG-wide patterns or distinct areas of zoning and hubs were revealed.

### Variations within model types

The distinction between zoned practice and hub models used was not as clear as indicated *.* Where hot hubs were co-located with cold services the requirement for strict management between hot and cold areas was as important as in zoned practices. Indeed, several CCGs reporting use of co-located hubs or zoned practices specified that separate entrances and exits were used, with some also reporting separate parking facilities. Other zoned practices meanwhile shared more similarities with distantly sited hubs, where red and green areas were split between main and branch surgeries for example, or where additional structures such as portacabins were used, to separate patient cohorts. Thus, it was not the case that the hub model always provided clearer separation and thereby simpler IPC adherence than zoned practices, as indicated in the guidelines. ^2, 4^

Use of dedicated home visits also varied. While within at least two CCGs this was the main or only form of COVID-19 face-to-face consultation, the use of home visits was not reported by 30% of CCGs. This may be due to the service being operated outside of primary care, as indicated by some. Home monitoring via pulse oximetry was also offered by general practice in a small number of CCGs during this period with, in one case, trained volunteers delivering the necessary equipment.

Further adaptations were shown by the temporary use of alternative non-healthcare settings and car-based models.

### Strengths and limitations

The study methodology used has both strengths and limitations. The use of a national survey, CCG level data collection and FOI requests will be considered in turn.

While this national survey has provided a picture of face-to-face general practice delivery in the first months of the pandemic in England, it no longer reflects current practice. Nevertheless, it enables review of early adaptations with the benefit of increased understanding of SARS-CoV-2 transmission, and of various impacts of the modified models on staff and patients. ^13- 16^ It may also be used to inform case site selection for more in-depth analysis to clarify issues such as those raised in this report, and to plan responses to further rises in incidence and new epidemics/pandemics.

The use of CCG level data facilitated a manageable national overview in an initial 6-week study. It is likely however that questioning at more local levels of organisation such as Primary Care Networks (groups of local GP practices serving populations of around 30,000 to 50,000 patients), ^17^ or at individual practice level would reveal greater nuance - in terms of model selection for example, practice size, staffing, local population and geographical factors may have been revealed to impact this, in addition to those indicated in our CCG responses. This may also be used to gain a better understanding of any distinct patterns of use within mixed models, and of home visiting services during the pandemic. Some CCG boundaries were also revealed to be somewhat flexible in terms of service delivery, with a degree of hub sharing reported. Close working was further indicated in some responses which were completed by one CCG on behalf of up to five others. The 2020 CCG mergers have already been discussed, and another round in April 2021 has since reduced numbers to 106. With all CCGs scheduled to merge across larger Integrated Care System boundaries in April 2022, a degree of complexity will be added to any future study utilising the current findings.

FOI requests, identified as a preferred data collection method by CCGs, ^18^ ensure high response rates within a mandated 4-week timeframe. They do not however readily permit further questioning, and both questions and responses may therefore be open to misinterpretation. While further investigation was used to minimize any error resulting from missing or ambiguous data, it is possible that models were misassigned in a small number of cases. [Data queries are noted in column J of the summary spreadsheet.] ^9^ An additional issue resulted from generic text within some responses requiring a further request to publish. While many CCGs agreed to this, different stipulations by others could not be met under the terms of the Open Access licence used and the data could not be published here.

## Conclusions

This study has provided an overview of adaptations to face-to-face GP consultations during the first four months of the COVID-19 pandemic in England. Varied and dynamic models were implemented to suit different and changing local conditions around the country. Evaluation of the delivery modifications described, including analysis of the management of both COVID-19 and other conditions, as well as other impacts on staff and patients, may also be used to identify beneficial elements of the rapidly enforced adaptations to inform practice both during the COVID-19 pandemic and beyond.

## Data Availability

The Re-use of Public Sector Information Regulations (RPSI) 2005 and copyright requirements have been invoked as imposing requirements around certain types of further use of survey data provided by some Clinical Commissioning Groups (CCGs). This may also apply to data received from other CCGs and it is therefore not possible to share this data under the terms of the Creative Commons Attribution 4.0 International license (CC-BY 4.0). The data may be available from individual CCGs on request, with reference to the authors and this publication. Alternatively, Freedom of Information requests similar to those made by the authors may be used. Full details of these are provided in the *Extended data* and *Methods* section. A summary spreadsheet of the authors' analysis of this data is also available: Figshare: Summary analysis of NHS face-to-face general practice models during the first wave of the COVID-19 pandemic in England March to July 2020, https://doi.org/10.6084/m9.figshare.14852517.v1. ^9^ Data are available under the terms of the Creative Commons Attribution 4.0 International license (CC-BY 4.0). Figshare: Survey sent to Clinical Commissioning Groups in England, https://doi.org/10.6084/m9.figshare.14156741.v1. ^6^ Data are available under the terms of the Creative Commons Attribution 4.0 International license (CC-BY 4.0).
