# Supplementary material for: Modifications to the delivery of NHS face-to-face general practice consultations during the COVID-19 pandemic in England
Source: F1000Res. 2021 Nov 1;10:261. Originally published 2021 Mar 31. [Version 3] doi: 10.12688/f1000research.52161.3 (PMC8611471; doi:10.12688/f1000research.52161.3)
Supplement: Peer review history

# Peer review: Reviewer response for version 3

**DOI:** 10.5256/f1000research.78362.r98364

**Published:** 2021-11-23

**Authors:** Mona Jeffreys

**Affiliations:** 1 Health Services Research Centre, Wellington Faculty of Health, Victoria University of Wellington, Wellington, New Zealand

I am happy to amend my recommendation to Approved, given the authors’ comments.

Is the work clearly and accurately presented and does it cite the current literature?

Yes

If applicable, is the statistical analysis and its interpretation appropriate?

Partly

Are all the source data underlying the results available to ensure full reproducibility?

No source data required

Is the study design appropriate and is the work technically sound?

Partly

Are the conclusions drawn adequately supported by the results?

Partly

Are sufficient details of methods and analysis provided to allow replication by others?

Partly

Reviewer Expertise:

NA

I confirm that I have read this submission and believe that I have an appropriate level of expertise to confirm that it is of an acceptable scientific standard.

# Peer review: Reviewer response for version 3

**DOI:** 10.5256/f1000research.78362.r98363

**Published:** 2021-11-01

**Authors:** Helen Atherton

**Affiliations:** 1 Warwick Medical School, Coventry, UK

No further comments.

Is the work clearly and accurately presented and does it cite the current literature?

Yes

If applicable, is the statistical analysis and its interpretation appropriate?

Partly

Are all the source data underlying the results available to ensure full reproducibility?

No source data required

Is the study design appropriate and is the work technically sound?

Yes

Are the conclusions drawn adequately supported by the results?

No

Are sufficient details of methods and analysis provided to allow replication by others?

Partly

Reviewer Expertise:

Access to general practice, remote consultation

I confirm that I have read this submission and believe that I have an appropriate level of expertise to confirm that it is of an acceptable scientific standard.

# Peer review: Reviewer response for version 2

**DOI:** 10.5256/f1000research.58863.r94410

**Published:** 2021-09-27

**Authors:** Mona Jeffreys

**Affiliations:** 1 Health Services Research Centre, Wellington Faculty of Health, Victoria University of Wellington, Wellington, New Zealand

This report describes changes that were implemented by general practice in England during the early months of the COVID-19 outbreak (i.e. March to July 2020).

 I am not clear about the lack of distinction regarding CCGs and practices. Some of the wording implies they are interchangeable terms (e.g. "The use of ‘co-located’ hubs was indicated in 21 CCGs, whereby hot hubs were sited adjacent to cold hubs (n = 4) or cold practices (n = 17)." Does this imply that within one CCG, all practices used the same model? If so, could this be stated explicitly? Figure 1 is equally confusing, with n=135 CCGs but n=12 "zoned practices".

 Figure 1 is not clear. I suggest that changes are made to the text below coloured circles in the top right pane, so that it is clear (if I have understood this correctly) that of 12 practices that used zones, six also used hot and/or cold home visits. Although category 7 has only one practice (or CCG?) , it would be helpful to include a circle for this, for consistency with Figure 2.

 It is not immediately clear to me how the data collected in the questionnaire resulted in the framework/ classification in Figure 1. In particular, what decisions/classifications were made by the authors to come up with the seven categories presented.

 I am unclear of the relevance of the data presented on clusters/outbreaks in GP practices. The inclusion of this data in this report implies that there is a relationship between the model of care that the CCG implemented, and an outbreak at a practice (reported on a regional, not CCG level). This is adequately dealt with in the paragraph entitled "SARS-CoV-2 transmission", but I do feel that maybe the data should not be included in the methods and results section. The statement "25 COVID-19 outbreaks linked to general practice were reported nationally in this period." should be removed from the conclusion as this is a statement of results, and implies that the conclusion is based on this. 

Is the work clearly and accurately presented and does it cite the current literature?

Yes

If applicable, is the statistical analysis and its interpretation appropriate?

Partly

Are all the source data underlying the results available to ensure full reproducibility?

No source data required

Is the study design appropriate and is the work technically sound?

Partly

Are the conclusions drawn adequately supported by the results?

Partly

Are sufficient details of methods and analysis provided to allow replication by others?

Partly

Reviewer Expertise:

Equity in access to primary health care

I confirm that I have read this submission and believe that I have an appropriate level of expertise to confirm that it is of an acceptable scientific standard, however I have significant reservations, as outlined above.

# Response

**Published:** 2021-10-28

**Authors:** Lorna Duncan

**Affiliations:** University of Bristol, UK

Dear Dr Jeffreys,

 Many thanks for reviewing our paper and for your helpful comments. We have now used these to improve our manuscript as indicated below in our responses to each point:

 1 *. I am not clear about the lack of distinction regarding CCGs and practices. Some of the wording implies they are interchangeable terms (e.g. "The use of ‘co-located’ hubs was indicated in 21 CCGs, whereby hot hubs were sited adjacent to cold hubs (n = 4) or cold practices (n = 17)." Does this imply that within one CCG, all practices used the same model? If so, could this be stated explicitly? Figure 1 is equally confusing, with n=135 CCGs but n=12 "zoned practices".*

 Thank you for highlighting this possibility for misinterpretation. We have now clarified the relationship between CCGs and GP practices in our Introduction. We have also updated the entire manuscript to ensure clearer distinction is made between GP practices and the CCGs they form part of wherever these terms are used. This includes, for example, greater clarity in Figure 1 that it was 12 of the 135 CCGs which were assigned to model #1, in which zoned practices (but not hubs) were used by their component GP practices. In relation to whether the models used by practices within any one CCG were common or heterogenous, we have also now clarified this both in the expanded *Data analysis* section in the Methods (please see also our response to your point 3 below); in the *Adapted delivery models* section, and the Figure 1 legend, in the Results; as well as in the *Model use* and *Strengths and limitations* sections in the Discussion.

 2. *Figure 1 is not clear. I suggest that changes are made to the text below coloured circles in the top right pane, so that it is clear (if I have understood this correctly) that of 12 practices that used zones, six also used hot and/or cold home visits. Although category 7 has only one practice (or CCG?) , it would be helpful to include a circle for this, for consistency with Figure 2.*

 Many thanks for highlighting the potential for confusion here. We have now expanded the Figure 1 legend to show that both model combinations 4 and 7 included home visits. We had not included them originally as these 2 models necessarily used home visits, but absolutely agree with you that it is clearer to show all 7 models in the top right pane. We have also included detail in the same legend to indicate that reference is being made to the CCGs rather than the GP practices. Fuller information  about the decisions regarding the inclusion of home visiting services in our model classifications has also now been given in the manuscript (please see also item 3 below).

 3. *It is not immediately clear to me how the data collected in the questionnaire resulted in the framework/ classification in Figure 1. In particular, what decisions/classifications were made by the authors to come up with the seven categories presented.*

 Thank you for this point, we see now that this was not explained sufficiently in our Methods section and have added detail (in the *Data analysis* section) describing how we arrived at the seven model types used to classify CCGs in this study. This includes discussion around the inclusion of home visiting services within these models, related to differences in the ways in which they were reported. Clarification regarding the use of home visits in the different models has also been added to the top right pane in Figure 1.

 4. *I am unclear of the relevance of the data presented on clusters/outbreaks in GP practices. The inclusion of this data in this report implies that there is a relationship between the model of care that the CCG implemented, and an outbreak at a practice (reported on a regional, not CCG level). This is adequately dealt with in the paragraph entitled "SARS-CoV-2 transmission", but I do feel that maybe the data should not be included in the methods and results section. The statement "25 COVID-19 outbreaks linked to general practice were reported nationally in this period." should be removed from the conclusion as this is a statement of results, and implies that the conclusion is based on this.*

 In light of your comments, it is clear that the outbreaks data is continuing to distract from the focus of our report. It is helpful to know this, thank you, and we have now removed each related section, as well as the Table.

 Many thanks for each of the points you have raised, we feel they have been very useful in enabling us to further improve this report.

# Peer review: Reviewer response for version 2

**DOI:** 10.5256/f1000research.58863.r92493

**Published:** 2021-08-23

**Authors:** Helen Atherton

**Affiliations:** 1 Warwick Medical School, Coventry, UK

I am happy with the changes, thank you. I have no further comments.

Is the work clearly and accurately presented and does it cite the current literature?

Yes

If applicable, is the statistical analysis and its interpretation appropriate?

Partly

Are all the source data underlying the results available to ensure full reproducibility?

No source data required

Is the study design appropriate and is the work technically sound?

Yes

Are the conclusions drawn adequately supported by the results?

No

Are sufficient details of methods and analysis provided to allow replication by others?

Partly

Reviewer Expertise:

Access to general practice

I confirm that I have read this submission and believe that I have an appropriate level of expertise to confirm that it is of an acceptable scientific standard.

# Peer review: Reviewer response for version 1

**DOI:** 10.5256/f1000research.55399.r83182

**Published:** 2021-05-04

**Authors:** Helen Atherton

**Affiliations:** 1 Warwick Medical School, Coventry, UK

This is a useful and clear report summarising the modifications made in general practice during the height of the pandemic.

 There are a few key things that would strengthen the manuscript particularly in terms of message.

- The abstract background says the study looked at 'evidence for their effectiveness' which is an overstatement as this is very much a descriptive study. It would be more accurate to remove that. 
- At present the paper is written as though we are still 'in' this stage of the pandemic. With vaccinations and the benefit of time changes are happening. It would be useful to couch the study as a description of service delivery in a time of crisis. Hot hubs are not going to be used in the same way now rates are extremely low. Your report is a really useful for any future lockdowns/rise in rates and as a learning tool and you don't couch it in those terms at present. 
- I completely appreciate why you used survey methodology but it isn't ideal as you later highlight. Using FOI requests is such a good idea but does make this a slightly different approach, and probably worth discussing pros/cons in the discussion for the benefit of other researchers. I was interested as to whether it is worth it for the data you get.
- The data analysis section is very thin and would benefit from some extra detail.
- The PHE element is probably the weakest bit, the link between the models and the outbreaks is tenuous as you don't have any other data around what happened there. Where you describe the 25 outbreaks it would be useful to have more context in the text - how many cases, which models, where. If this is not available I think this needs to be highlighted.
- There are several limitations to be added to the discussion  - for example the bit about the CCG mergers, which is important. 
- The section in the discussion about transmission is useful context but comes a bit late in terms of understanding that bit of the work. 
- The section on evaluation doesn't add anything at this point. Could you say instead what the practical application of your findings might be? 
- Overall the manuscript lacks a bit of context, which is fine if it is just a descriptive exercise, but at the moment it isn't sure if it is something more than that. If it is a useful descriptive tool that is fine, but the conclusion probably needs to be toned down. In particular the line 'indicate their relative success in minimising transmission' - you just don't have the data to say that.
- Figure is really good and the succinct messaging was appreciated. 

Is the work clearly and accurately presented and does it cite the current literature?

Yes

If applicable, is the statistical analysis and its interpretation appropriate?

Partly

Are all the source data underlying the results available to ensure full reproducibility?

No source data required

Is the study design appropriate and is the work technically sound?

Yes

Are the conclusions drawn adequately supported by the results?

No

Are sufficient details of methods and analysis provided to allow replication by others?

Partly

Reviewer Expertise:

Access to general practice

I confirm that I have read this submission and believe that I have an appropriate level of expertise to confirm that it is of an acceptable scientific standard, however I have significant reservations, as outlined above.

# Response

**Published:** 2021-08-20

**Authors:** Lorna Duncan

**Affiliations:** University of Bristol, UK

Dear Dr Atherton,

 Many thanks for taking the time to review this paper. We have used your detailed and helpful comments to improve our manuscript. Our specific responses to each of your points are listed below.

**Key points to strengthen the manuscript:**

 1. *The abstract background says the study looked at 'evidence for their effectiveness' which is an overstatement as this is very much a descriptive study. It would be more accurate to remove that.*

 We have removed this sentence further to your feedback. Please see also our response to your point 5 below.

 2. *At present the paper is written as though we are still 'in' this stage of the pandemic. With vaccinations and the benefit of time changes are happening. It would be useful to couch the study as a description of service delivery in a time of crisis. Hot hubs are not going to be used in the same way now rates are extremely low. Your report is a really useful for any future lockdowns/rise in rates and as a learning tool and you don't couch it in those terms at present.*

 We have now ensured the entire manuscript is written from a historic perspective, looking back to the first wave of the pandemic in spring and early summer 2020. We wrote this report during the second wave as incidence fluctuated, and we had difficulty deciding how to contextualise it. We are grateful for your perspective and agree that this revision has given a much clearer context for the findings. 

 3. *I completely appreciate why you used survey methodology but it isn't ideal as you later highlight. Using FOI requests is such a good idea but does make this a slightly different approach, and probably worth discussing pros/cons in the discussion for the benefit of other researchers. I was interested as to whether it is worth it for the data you get.*

 We have now included further discussion of the pros and cons of the use of both surveys and FOI requests in the new ‘strengths and limitations’ section. We were somewhat limited in the design of our study as COVID-19 restrictions required it to be completed remotely, and because it was initially intended as a 6-week project. Despite the limitations however, we feel this survey using FOI requests yielded useful information, either for case site selection and further investigation, or for retrospective analysis for initial management of any future epidemics, or future lockdowns as you suggest. Although it was not possible to include a comparative table of the CCG data received, we hope that the additional information now included in our summary analysis table (as *Underlying data*) supports this and is found useful. Please see also our response to your point 8 below.

 4. *The data analysis section is very thin and would benefit from some extra detail.*

 We have now incorporated more detail in our methodology section and have also added a summary version of our analysis spreadsheet as *Underlying data*. It is unfortunate that we are unable to include copies of the CCG and PHE responses under the terms of the Creative Commons Attribution 4.0 International license (CC-BY 4.0) used in this article. However, on considering your comments, we realised it may be helpful to offer permissible detail from which figures 1 and 2 were derived by adding a version of our analysis spreadsheet with the raw data removed. Of course this includes only an interpretation of the data received.

 5. *The PHE element is probably the weakest bit, the link between the models and the outbreaks is tenuous as you don't have any other data around what happened there. Where you describe the 25 outbreaks it would be useful to have more context in the text - how many cases, which models, where. If this is not available I think this needs to be highlighted.*

 We agree that the data we have concerning general practice-linked COVID-19 outbreaks is limited and we are grateful to you for highlighting the possible inference of a direct link between the models used and outbreaks from the text. This was not our intention and we have now given a fuller account of the reasoning for requesting this data and for including it in our report. We have also separated this work out in each section of the report.

 To the best of our knowledge, this data has not otherwise been made publicly available and we feel it offers useful context around the scale of outbreaks in general practice. We requested further information both from PHE and NHS England & NHS Improvement and have now mentioned this in our methodology section and reported the finding that transmission in staff was more common in general practice than that involving patients in one region. PHE was not able to provide more detail on specific outbreaks due to the potential for deductive disclosure however, and the table we have included in our report provides the most detailed data made available to us.

 6. *There are several limitations to be added to the discussion - for example the bit about the CCG mergers, which is important.*

 We have now expanded on our limitations section, to include discussion around the CCG mergers and several other items. Please see our response to item 8 below for more information.

 7. *The section in the discussion about transmission is useful context but comes a bit late in terms of understanding that bit of the work.*

 We agree and thank you for highlighting this. We have now moved this section to the introduction and expanded it.

 8. *The section on evaluation doesn't add anything at this point. Could you say instead what the practical application of your findings might be?*

 [Combined response for items 3, 6 and 8]. We have replaced both the ‘evaluation’ and ‘limitations’ sections with a fuller ‘strengths and limitations’ section. This includes further discussion of the survey methodology, using FOI requests at CCG level, the CCG mergers and possible applications of our study. We agree that this is likely to be far more useful than the two previously included sections – many thanks.

 9. *Overall the manuscript lacks a bit of context, which is fine if it is just a descriptive exercise, but at the moment it isn't sure if it is something more than that. If it is a useful descriptive tool that is fine, but the conclusion probably needs to be toned down. In particular the line 'indicate their relative success in minimising transmission' - you just don't have the data to say that.*

 This report is intended to offer a description of the face-to-face services offered in general practice in the initial phases of the pandemic. The information we included concerning outbreaks in general practice perhaps confused this although we do feel it adds some useful context to our data. We have now removed the line you mention and agree that our conclusion now more clearly represents our study.

 10. *Figure is really good and the succinct messaging was appreciated.*

 Many thanks, we are pleased you found this helpful! Thank you very much also for each of your comments which we feel have helped us to improve this report.
